# Treatment of coronary lesions with a novel crystalline sirolimus-coated balloon

**DOI:** 10.3389/fcvm.2024.1316580

**Published:** 2024-02-13

**Authors:** Mehdi Madanchi, Adrian Attinger-Toller, Varis Gjergjizi, Irena Majcen, Giacomo M. Cioffi, Angelika Epper, Eleonora Gnan, Tanja Koch, Yuan Zhi, Florim Cuculi, Matthias Bossard

**Affiliations:** ^1^ Cardiology Division, Heart Center, Luzerner Kantonsspital, Lucerne, Switzerland; ^2^ Department of Health Sciences and Medicine, University of Lucerne, Lucerne, Switzerland; ^3^ McMaster University, Hamilton, ON, Canada; ^4^ Hamilton Health Sciences, Hamilton, ON, Canada; ^5^ Faculty of Medicine, University of Basel, Basel, Switzerland; ^6^ Fondazione IRCCS Cà Granda Ospedale Maggiore Policlinico di Milano, University of Milan, Milan, Italy

**Keywords:** drug-coated balloons, sirolimus, paclitaxel, complex coronary lesions, percutaneous coronary intervention, drug eluting stent

## Abstract

**Background:**

There is mounting data supporting the use of drug-coated balloons (DCB) not only for treatment of in-stent restenosis (ISR), but also in native coronary artery disease. So far, paclitaxel-coated balloons represented the mainstay DCBs. The SeQuent® crystalline sirolimus-coated balloon (SCB) (B.Braun Medical Inc, Germany) represents a novel DCB, which allows a sustained release of the limus-drug. We evaluated its performance in an all-comer cohort, including complex coronary lesions.

**Methods:**

Consecutive patients treated with the SeQuent® SCB were analyzed from the prospective SIROOP registry (NCT04988685). We assessed clinical outcomes, including major adverse cardiovascular events (MACE), target lesion revascularization (TLR), target vessel myocardial infarction (TV-MI) and cardiovascular death. Angiograms and outcomes were independently adjudicated.

**Results:**

From March 2021 to March 2023, we enrolled 126 patients and lesions, of which 100 (79%) treated using a “DCB-only” strategy and 26 (21%) with a hybrid approach (DES + DCB). The mean age was 68 ± 10 years, 48 (38%) patients had an acute coronary syndrome. Regarding lesion characteristics, ISR was treated in 27 (21%), 11 (9%) underwent CTO-PCI and 59 (47%) of the vessels were moderate to severe calcified. Procedural success rate was 100%. At a median follow-up time of 12.7 (IQR 12; 14.2) months, MACE occurred in 5 patients (4.3%). No acute vessel closure was observed.

**Conclusions:**

Our data indicates promising outcomes following treatment with this novel crystalline SCB in an all-comer cohort with complex coronary lesions. These results require further investigation with randomized trials.

## Introduction

Drug-coated balloons (DCBs) constitute a rather novel treatment approach for coronary artery disease (CAD), which lately gained an increasing popularity following a series of landmark trials indicating their efficacy in treatment of in-stent restenosis (ISR) and native CAD (1–4). The lipophilic matrix of DCBs ensures a homogenous transfer of antiproliferative drugs into the vascular wall, which often permits omitting implantation of a drug eluting stent (DES) and thus leaving the vessel free of any permanent metallic implants (5).

Albeit a variety of DCB catheters and additives for drug transfer have been introduced over the last decade, paclitaxel, which reflects a potent antimitotic drug targeting tubulin, still is the drug of choice for most DCB manufacturers (5).

Following the successful introduction of limus-eluting stent platforms, there has been an ongoing interest to investigate limus-based drugs for the application in DCBs. However, this process has been rather challenging and several technical hurdles had to be overcome, since limus-based drugs, including sirolimus, show a low acute absorption and transfer rate due to the drugs rather low lipophilicity, in comparison to paclitaxel (6, 7).

The SeQuent® sirolimus-coated balloon (SCB) (B.Braun Medical Inc., Germany) represents a novel DCB comprising a crystalline sirolimus coating, which allows a slow and persistent release of the drug within the vessel wall up to 1 month after application (8). Early randomized studies including only selected patients indicated good results in ISR and native lesions following treatment with this SCB (9–11).

Since real-world data about the safety and performance of this crystalline SCB is lacking, we assessed real-world outcomes from an all-comer CAD cohort, including also complex characteristics such as chronic total occlusions (CTO) and ISR lesions, which has been treated with this novel DCB device.

## Methods

**Figure 1:**
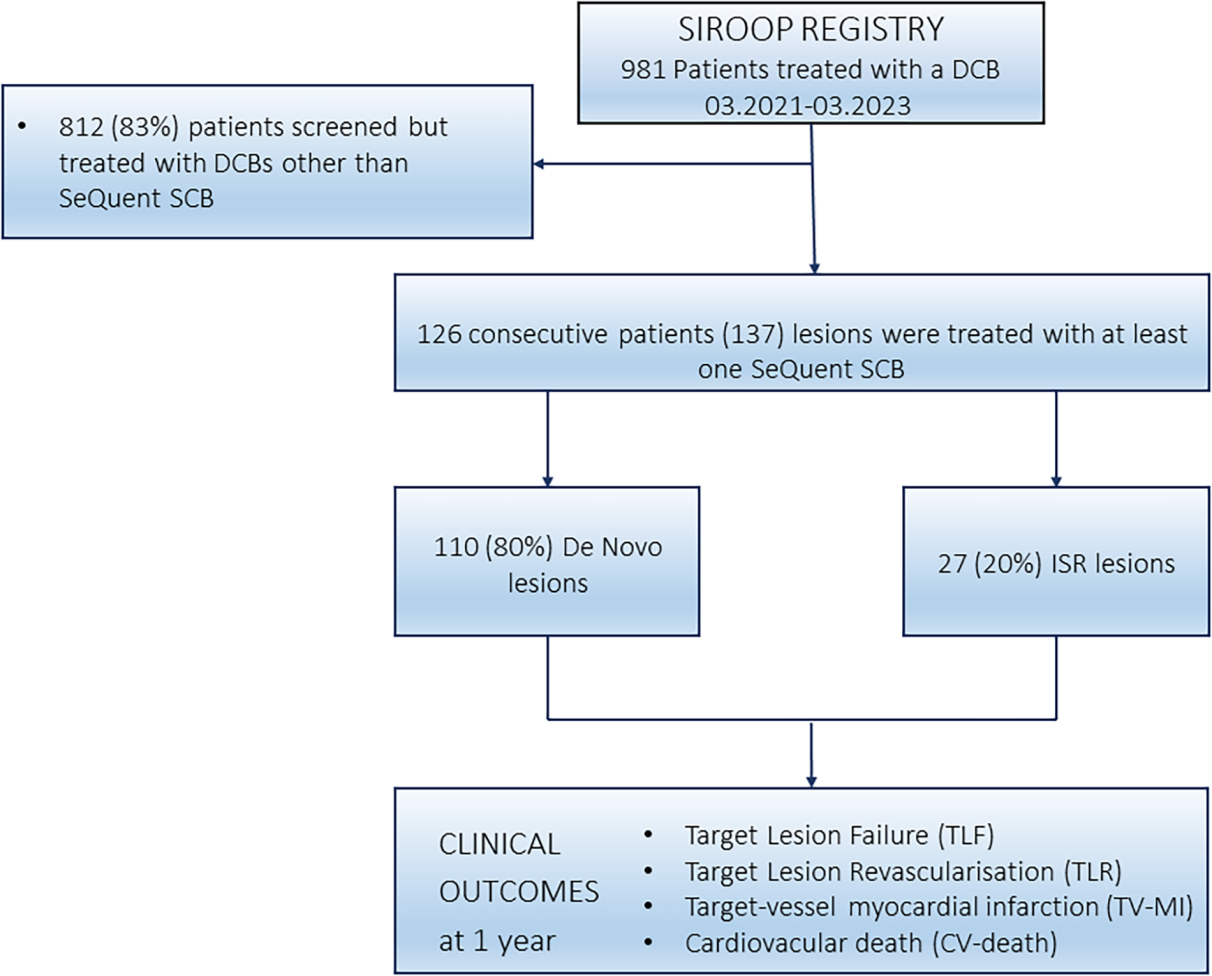
Study flow chart.

We analyzed consecutive patients from the prospective *SIROOP Registry (Prospective Registry Study to Evaluate the Outcomes of Coronary Artery Disease Patients Treated With SIROlimus Or Paclitaxel Eluting Balloon Catheters)* (ClinicalTrials.gov identifier: NCT04988685), which was designed to assess the management and outcomes of patients with acute (ACS) and chronic coronary syndrome (CCS) undergoing percutaneous coronary interventions (PCI) with contemporary DCBs. For this study, we analyzed patients who have been treated with the SeQuent® SCB at the Heart Center of the Lucerne Cantonal Hospital (Lucerne, Switzerland), the tertiary cardiology facility for the central part of Switzerland. Figure 1 depicts the study flow chart.

### The study device

**Figure 2:**
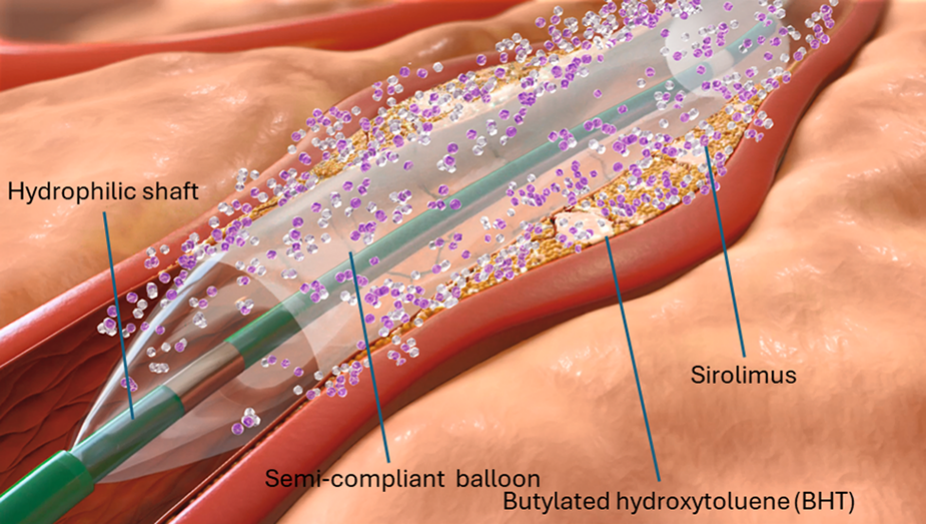
Illustration depicting the specific characteristics of the crystalline seQuent® SCB (We used this illustration with friendly permission of braun medical Inc., Germany).

The SeQuent® SCB´s coating consists of a crystalline sirolimus formulation (12). It uses butylated hydroxytoluene (BHT) as an excipient, which allows a sustained release of the drug as depicted in Figure 2 (5, 12). The sirolimus drug concentration is 4 μg/mm^2^ balloon surface (12). The system consists in hydrophilic coated surface and a low-tip profile semi-compliant coronary. Balloon sizes are available from 2.0 to 4.0 mm in diameter and 10–40 mm in length. The recommended inflation time is at least 30 s and a balloon length exceeding 2–3 mm (proximally and distally) the predilated segment is recommended.

### Study population

Consecutive patients with both ACS and CCS undergoing PCI with at least one SeQuent® SCB were analyzed from the SIROOP registry. Of note, we applied no angiographic exclusion criteria, meaning with also considered complexe coronary lesions (e.g., bifurcation, calcified and CTO and ISR) for this analysis.

From every study participant, demographic and procedural data were collected using a dedicated database (REDCap^©^, Version 10.6.28, established by the Vanderbilt University, Tennessee, U.S.A.). Follow-up was obtained by clinic visits or telephone interviews at 6 and 12 months following the procedure.

### PCI procedure

PCI procedures were conducted in accordance with the international practice guideline recommendations (13–15). When using DCBs, our internal practice recommendations support the liberal utilization of intravascular imaging (intravascular ultrasound or optical coherence tomography) to plan lesion preparation and DCB sizing. All interventionalists involved in this study almost routinely use cutting balloons and/ or non-compliant (NC) for lesion preparation (16). This is conform to the 3rd DCB consensus paper (5). Following successful lesion preparation, and in the absence of major complications (e.g., >30% residual stenosis, flow limiting dissections or menacing abrupt vessel closure), at least one SeQuent® SCB was applied to the target lesion. The devices were used according to the manufactureŕs instructions for use. Whenever possible, we tried to inflate the SCB for at least 45 s, to allow optimal drug transfer to the treated vessel segments. In DCB cases, bailout stenting was defined as necessity to place a DES in order to ensure vessel patency and/or restore flow following treatment with the SCB. We defined as hybrid approach, lesions which needed direct implantation of DES following lesion preparation (e.g., due to recoil or deep dissections) and the rest of the vessel were treated with SCBs.

### Antithrombotic treatment

Considering the applied antithrombotic regimens, we followed the current guidelines (5, 13, 14). Patients received aspirin prior to PCI and were then loaded with thienopyridines, at the discretion of the treating physician with either clopidogrel, ticagrelor or prasugrel, during or immediately after the PCI. Heparin was administered at a dosing of 70–100 units/kilogram body weight aiming for a target ACT >250 s during PCI. The duration of DAPT varied between 1 and 3 months in case of DCB-only treatment, which is in line with the Third Report of the International DCB Consensus Group and the patient's bleeding and thrombotic risk (5). In case a DES was implanted the suggested duration of DAPT ranged from 6 to 12 months, according to the latest guidelines (13). In patients with an indication for anticoagulation, we recommended the use of a direct oral anticoagulant in combination with aspirin for maximally 1 week in combination with clopidogrel for 1–12 months.

### Angiographic analyses

Two independent physicians not involved in the procedure (MM and GMC) analyzed the angiograms with a dedicated software package (Intellispace cardiovascular, Phillips, Koninklijeke, Netherlands). The calcium was scored based on the three-tier classification system: Minimal or no calcification; calcium covering ≤50% of the circumference of the vessel is classified as moderate calcification; calcium covering 50%–100% of the circumference of the vessel is classified as severe calcification. Classification of dissections was performed according to the National Heart, Lung and Blood Institute (NHLBI) classification system for intimal tears, consisting of Type A through Type F (17).

### Study outcomes

Clinical outcomes of interest included major adverse cardiovascular events (MACE), defined as composite of cardiac death, target vessel myocardial infarction (TV-MI) and target lesion revascularization (TLR). Other outcomes of interest included target vessel revascularization (TVR) and all-cause death. For definitions, we followed the criteria of the Academic Research Consortium (ARC) (18). We also collected detailed information on any periprocedural complications [e.g., coronary perforations, urgent coronary artery bypass grafting (CABG), bleedings and strokes]. Procedural success was defined as a residual stenosis of <30% remaining after PCI with a TIMI flow grade 3 at the end of the procedure and freedom from any major procedure-related complication. All outcomes were independently adjudicated by two experienced physicians not involved in the procedures (MM and GMC).

### Statistical analysis

Categorical variables were presented as frequencies and percentages, while continuous variables were displayed as means (±standard deviations) or medians [interquartile ranges (IQR)], as appropriate. In our analyses of continuous variables, inspection of the distribution patterns was used to determine the appropriate statistical test. *P*-values were calculated using Fisher's exact test, *t*-test, and Wilcoxon rank-sum test, and were adjusted using the Benjamini and Hochberg method. For outcome variables, we employed Gray's test. Outcomes over time were plotted using Kaplan–Meier curves. A two-tailed *p*-value of <0.05 was considered statistically significant. Data analysis was conducted using R Statistical Software (v4.2.2; R Core Team, 2022).

## Results

### Patient and lesion characteristics

Overall, 126 patients were included, who have been treated with SeQuent® SCBs between March 2021 and March 2023 at our institution, as highlighted in Figure 1. Patients were mainly males, and approximately one third of patients presented with an ACS. Table 1 summarizes the details on baseline characteristics.

**Table 1:** Baseline characteristics of the study population.

	Overall (*n* = 126)	De novo (*n* = 99)	ISR (*n* = 27)	*p*-value
Age (years)	68 ± 10	68 ± 10	68 ± 11	1.0
Males (%)	108 (86)	88 (89)	20 (74)	0.4
Presentation (%)				
CCS	78 (62)	57 (58)	21 (78)	0.8
ACS	48 (38)	42 (42)	6 (22)	0.09
NSTEMI	24 (19)	21 (24)	6 (22)	0.4
STEMI	6 (5)	6 (6)	0 (0)	0.4
Cardiovascular risk factors (%)				
Arterial hypertension	92 (73)	70 (71)	22 (81)	0.7
Diabetes mellitus	39 (31)	28 (28)	11 (41)	0.6
Dyslipidemia	104 (82)	79 (80)	25 (93)	0.6
Current smoking	19 (15)	15 (15)	4 (15)	1.0
Previous MI (%)	54 (43)	39 (39)	15 (56)	0.6
Previous CABG (%)	12 (9.5)	6 (6.1)	6 (22)	0.2
HF (%)	17 (13)	15 (15)	2 (7.4)	0.8
Antithrombotics (%)				
Aspirin	109 (87)	86 (87)	23 (85)	1.0
Clopidogrel	58 (46)	46 (46)	12 (44)	1.0
Ticagrelor	31 (25)	24 (24)	7 (26)	1.0
Prasugrel	27 (21)	22 (22)	5 (18)	1.0
Oral anticoagulant	16 (13)	14 (14)	2 (7.4)	0.8

Data are mean (standard deviation), median (interquartile range) or number (percentage), as appropriate.

ACS, Acute coronary syndrome; CABG, Coronary artery bypass grafting; CCS, Chronic coronary syndrome; HF, heart failure with reduced ejection fraction (LVEF <40%); ISR, in-stent restenosis; MI, myocardial infarction; STEMI, ST-segment elevation myocardial infarction; NSTEMI, non-ST segment elevation myocardial infarction; No., number.

Totally, 126 lesions were treated with at least one SeQuent® SCB. Of these, 100 (79%) lesions were managed with a DCB only and 26 (21%) with a hybrid strategy. Most lesions involved the left anterior descending artery 51 (41%) and left circumflex artery 41 (32%). ISR was present in 27 (21%) patients, 11 (9%) had a CTO-PCI and 59 (47%) of the lesions were moderately to severely calcified.

**Figure 3:**
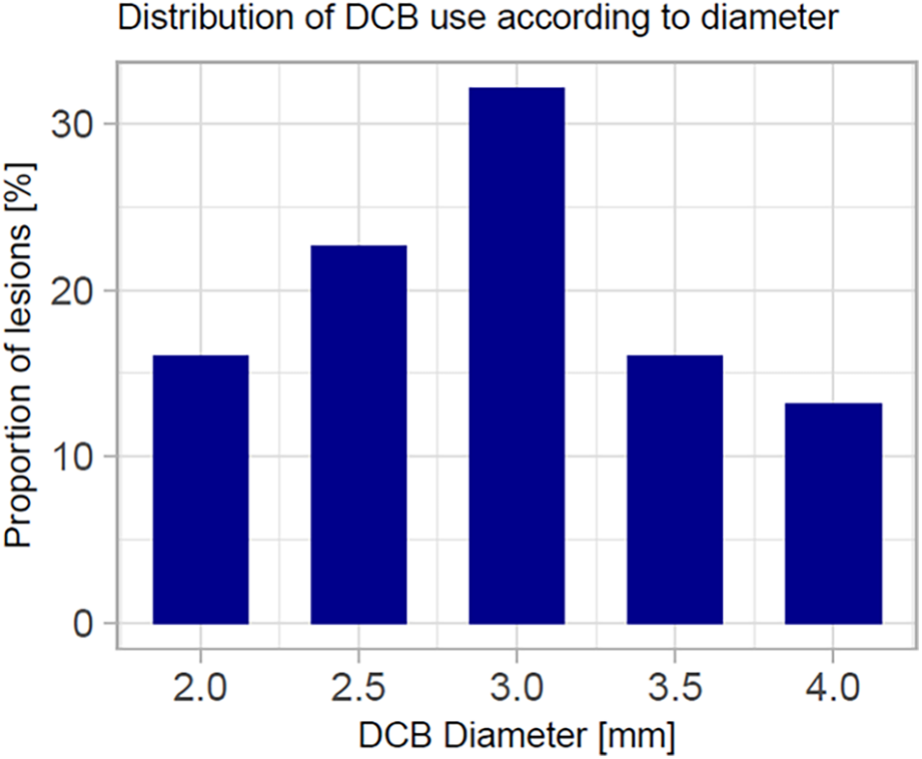
Diagram depicting the frequency of each DCB used according to its diameter.

**Figure 4:**
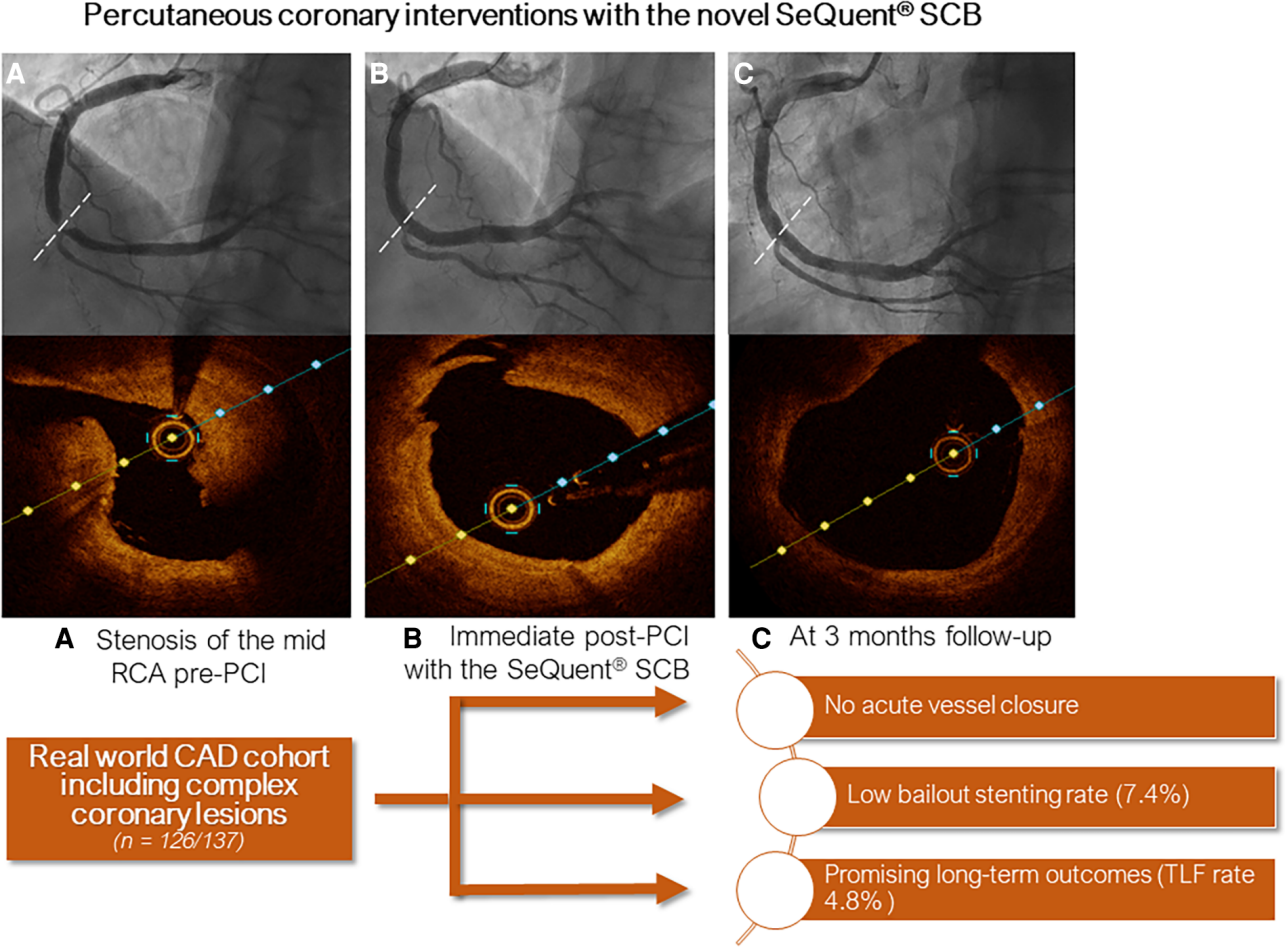
Central figure depicting an illustrative case of a patient treated with the crystalline seQuent® SCB. (**A**) Angiogram and optical coherence tomography (OCT) at index procedure showing stenosis of the mid right coronary artery; (**B**) Immediate post-PCI and (**C**) at 3 months follow-up.

Lesion preparation was predominately performed with super non-compliant balloons (88%) inflated at high-pressure (mean inflation pressure was 29 ± 9atm), whereas cutting balloons were used in a total of 88 lesions (70%). The mean SCB diameter was 2.9 ± 0.6 mm and mean inflation pressure was 6 ± 3atm. At index procedure, we encountered 3 (2.1%) relevant dissections (D-F). Of note, all dissections occurred after deployment of the DCB. In 10 (8%) cases, bailout stenting was necessary. Further procedural characteristics are reported in Table 2. Figure 3 depicts the percentage of DCB used according to their diameter. In Figure 4, we depicted an illustrative case of a patient presenting with NSTEMI, which has been successfully treated using a SeQuent® SCB.

**Table 2:** Lesion and procedural characteristics of the study population.

	Overall (*n* = 126)	De novo (*n* = 99)	ISR (*n* = 27)	*p*-value
Access (%)				
Radial	101 (80)	81 (82)	20 (74)	0.4
Femoral	25 (20)	18 (18)	7 (26)	0.6
Vessel treated (%)				
Left anterior descending artery	51 (41)	42 (42)	9 (33)	0.9
Left circumflex artery	41 (32)	36 (36)	5 (19)	0.5
Right coronary artery	34 (27)	19 (19)	15 (56)	0.2
SYNTAX score	15 ± 10	16 ± 10	13 ± 9	0.5
ACC/AHA lesion classification (%)				0.6
Type B1	51 (40)	41 (41)	10 (37)	
Type B2	38 (30)	33 (33)	5 (19)	
Type C	37 (30)	25 (26)	12 (44)	
Aorto-ostial lesions (%)	33 (26)	21 (21)	12 (44)	0.05
Bifurcation lesions (%)	45 (36)	37 (34)	8 (30)	0.9
Medina (1,1,1)	21 (17)	15 (15)	6 (22)	
Medina (1,1,0)	12 (9)	11 (11)	1 (4.0)	
Medina (0,1,1)	2 (2.0)	1 (1.0)	1 (4.0)	
CTO lesions (%)	11 (9)	10 (10)	1 (4.0)	0.9
Moderate to severe calcifications (%)	59 (47)	43 (43)	16 (59)	0.3
Type of pre-dilatation balloons (%)				
SC balloons	16 (13)	13 (13)	3 (11)	1.000
NC balloons	81 (64)	61 (62)	20 (74)	0.3
Super NC balloons	112 (89)	89 (90)	23 (85)	0.9
Cutting balloons	88 (70)	81 (82)	7 (26)	<0.001
IVL	10 (7.9)	4 (4.0)	6 (22)	0.03
Rotational atherectomy	1 (0.8)	0 (0)	1 (3.7)	0.5
Largest pre-dilatation balloon diameter (mm ± SD)	3.1 ± 0.6	3.0 ± 0.6	3.4 ± 0.1	0.06
Maximal pre-dilatation pressure (atm ± SD)	29 ± 9	28 ± 9	34 ± 9	0.01
DCB diameters (mm ± SD)	2.9 ± 0.6	2.8 ± 0.6	3.3 ± 0.6	0.01
DCB inflation pressure (atm ± SD)	6 ± 3	6 ± 2	9 ± 4	0.001
Mean number of DCB used (*n* ± SD)	1.7 ± 1	1.6 ± 0.9	1.9 ± 1.3	0.1
Intravascular imaging (%)				
OCT	57 (45)	43 (43)	14 (52)	0.8
IVUS	3 (2.4)	2 (2.0)	1 (3.7)	0.8
Dissections post-DCB (%)				0.9
Type A	0 (0)	0 (0)	0 (0)	
Type B	1 (0.8)	1 (1.0)	0 (0)	
Type C	7 (5.6)	6 (6.0)	1 (3.7)	
Type D	1 (0.8)	1 (1)	0 (0)	
Type E	1 (0.8)	1 (1)	0 (0)	
Type F	1 (0.8)	1 (1)	0 (0)	
Bailout stenting (%)	10 (8.0)	9 (9.1)	1 (3.7)	0.7

Data are mean (standard deviation) or number (percentage), as appropriate; CTO, chronic total occlusion; DCB, drug coated balloon; DES, drug eluting stents; IVUS, intravascular ultrasound; IVL, intravascular lithotripsy; ISR, in-stent restenosis; NC, non-compliant; No., number; OCT, optical coherence tomography; SC, semi-compliant balloon.

### Clinical outcomes

Clinical follow-up was obtained in 117 (93%) patients, with a median follow-up duration was 12.7 (IQR 12; 14.2) months. Within the first year, MACE occurred in 5 (4.3%) patients. Of them 3 patients required TLR for restenosis and 2 patients succumbed a cardiovascular death (1 patient suffered from acute heart failure and 1 patient presented with ACS complicated by cardiogenic shock).

**Figure 5:**
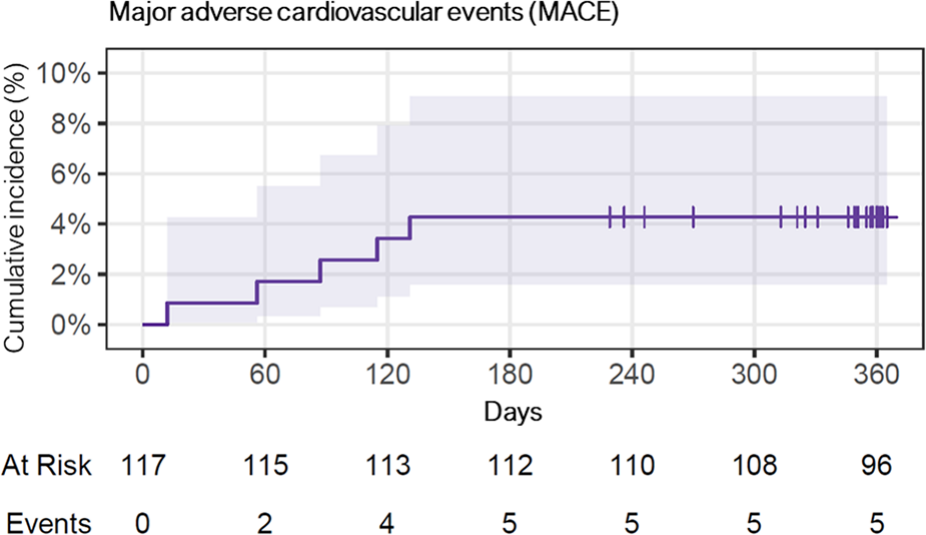
Cumulative incidence curve for major adverse cardiovascular events (MACE), of the whole population over 1 year. Of note, 7 patients were lost during follow-up, and 9 patients had follow-up between day 300–365.

**Figure 6:**
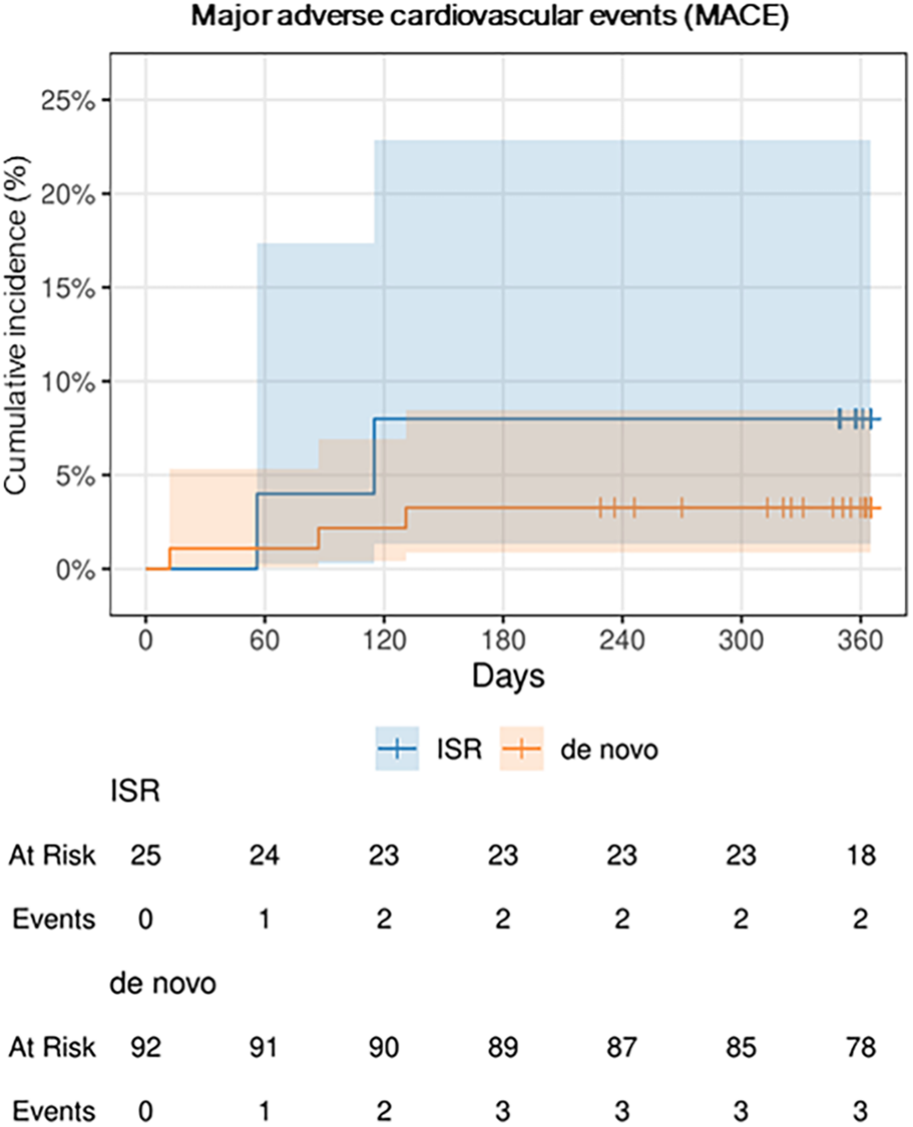
Cumulative incidence curve for major adverse cardiovascular events (MACE), the two subgroups “de novo” and in-stent restenosis (ISR) over 1 year.

The incidence of MACE was numerically, but not statistically significant higher in ISR lesions, with 2 events (8%), compared to *de novo* lesions, which had 3 events (3%). Further details can be found in Table 3, Figure 5 and Figure 6 depict the cumulative incidence curves for MACE across the entire population and within the de-novo and ISR subgroups treated with SCBs. Table 4 reports details about patients with a MACE. Of note, we encountered no clinically relevant bleeding events following index PCI and during follow-up.

**Table 3:** Clinical outcomes at 12 months follow-up.

	Overall^a^	De novo	ISR
Patients at follow-up (%)	117 (92)	92 (93)	25 (93)
MACE, *n* (%; 95% CI)	5 (4; 2–9)	3 (3; 1–9)	2 (8; 1–24)
* *TLR	2 (2; 0.5–5.5)	1 (1; 0.1–5)	1 (4; 0.3–19)
* *TV-MI	0	0	0
* *Cardiac death	3 (3; 1–7)	2 (2; 0.5–7)	1 (4; 0.3–18)
TVR, *n* (%; 95% CI)	0	0	0
All-cause death, *n* (%; 95% CI)	3 (3;1–7)	2 (0.5–7)	1 (4; 0.3–18)
CABG, *n* (%; 95% CI)	1 (1; 0.1–4)	1 (1; 0.1–5)	0
Thrombotic vessel closure, *n* (%; 95% CI)^b^	0	0	0
Stroke, *n* (%; 95% CI)	1 (1; 0.1–4)	1 (1; 0.1–5)	0

Data are presented as number (percentage) and represent cumulative event rates (with 95% confidence intervals).

CABG, coronary artery bypass grafting; ISR, in-stent restenosis; MACE, major adverse cardiac events; TLR, target lesion revascularization; TV-MI, target vessel myocardial infarction; TVR, target vessel revascularization; CI, confidence interval.

^a^ There were no significant differences between the groups.

^b^ This includes (acute) vessel closure following DCB treatment as well as any kind of stent thrombosis.

**Table 4:** Narratives of the patients presenting with major adverse cardiovascular events (MACE) during the follow-up period.

MACE no.	Time to MACE (days)	MACE presentation	Presumed cause of MACE	Indication for index PCI	Target vessel	SCB^a^ diameter (mm)	SCB^a^ length (mm)	P2Y12 inhibitor
1	20	UA	Restenosis^c^	CCS	Proximal RCA	4.0	30	Prasugrel
2	115	Silent ischemia^b^	Restenosis^c^	ACS (UA)	Mid LAD	2.0	40	Prasugrel
3	131	Silent ischemia^b^	Restenosis^c^	CCS	LM/Ostial-Distal LCX	3.0	40	Clopidogrel
4	56	CV death	–	CCS	Ostial-Distal LAD	4.0	40	Clopidogrel
5	12	CV death	–	CCS	LM/LAD/LCX	3.0	40	Clopidogrel

ACS, acute coronary syndrome; CCS, chronic coronary syndrome; CV death, cardiovascular death; SCB, crystalline sirolimus coated balloon; MACE, major adverse cardiovascular events; LAD, left anterior descending coronary artery; LCX, left circumflex coronary artery; LM, left main; PCI, percutaneous coronary intervention; RCA, right coronary artery; UA, unstable Angina.

^a^ This indicates the maximal diameter and length of crystalline sirolimus coated balloon applied at the index intervention.

^b^ Restenosis in all 3 cases was attributable to recoil.

^c^ Those 2 patients showed angiographically significant restenosis on follow-up angiograms.

## Discussion

This is one first reports summarizing clinical outcomes of a real-world CAD population treated with the novel crystalline SeQuent® SCB. One needs to take into account, that DCBs for treatment of *de novo*, and furthermore complex coronary lesions (e.g., calcified and CTO lesions), have not been broadly embraced yet. The use of this novel SCB for treatment of CAD seems not only to be safe, but also related to a low 1-year MACE rate (<5%). In addition, the outcomes appear not to significantly differ between native and ISR lesions treated with this SCB.

So far, this crystalline SCB has been compared to an established paclitaxel-coated balloon (PCB), the SeQuent® Please NEO, in two randomized trials, which focused on angiographic outcomes (9, 11). In these two studies no significant differences were found in both ISR and *de novo* lesions (9, 11). Although these results seem encouraging, both trials were also rather small and enrolled only well selected patients and lesions, which may limit the translation of those results into current clinical practice.

In this context, our results may not only expand those, but also the results from earlier randomized trials and real-world registries, which generally studied the safety and performance of paclitaxel-coated balloons (4, 19). Of note, most of those studies included relatively simple coronary lesions or focused on treatment of small coronary vessels (<3 mm diameter) (4, 19). Moreover, a lot of data supporting DCBs derives from studies assessing their utility in ISR treatment (2). Contrastingly, we have applied this crystalline SCB for treatment of highly calcified lesions (43%), bifurcations (33%), CTOs (8%) as well as ISR lesions. Also, a relevant group of our study cohort (79%) underwent treatment of native coronary lesions involving large and/or main coronary vessel segments with SCBs, again such cases have generally been underrepresented in previous reports (20).

With regards to clinical outcomes, we observed a lower MACE rate than earlier studies. For instance, the pivotal randomized BASKET-SMALL II and PICCOLETTO-II trials comparing PCBs to DES reported MACE rates of 7.5% and 5.6%, respectively (4, 19). When considering real world evidence, a study by Wöhrle and colleagues showed a TLR rate of 5.2% at 9 month follow-up with the SeQuent® Please PCB (21). However, this study included a large portion of BMS-ISR cases and excluded complex lesions (severely calcified, CTOs lesions) (21). When focusing on SCBs, a new study by Cortese et al. highlighted a MACE rate of 9.9% at 12 months with a recently approved DCB, the MagicTouch™ (Concept Medical Inc., Florida, U.S.A.), which uses phospholipid-based nanocarriers for sirolimus transfer (20). Interestingly, this study reported a relatively high rate of MACE in ISR lesions (14.9% vs. 4.9% in de-novo lesions) (20). In addition, we have recently published our early experience with another SCB, the Selution SLR™ (MedAlliance SA, Switzerland), in an all-comer CAD population, which had similar characteristics the current study cohort (16). There, the MACE rate at 1 year was slightly higher (6.8%).

In the actual study cohort, after a median time of 56 (IQR 16; 123) days, 5 (4.3%) patients suffered a MACE. Those patients showing TLR presented with rapidly worsening chest pain (unstable angina) in one case, and silent ischemia in two cases. Restenosis, which was mainly imputable to lesion recoil, was found in all 3 patients requiring TLR. Anyways, no patients needed urgent revascularization and no case of acute vessel closure was encountered. This seems reassuring and may indicate that in the absence or presence of only short segments of newly implanted DES the risk for acute vessel closure or stent thrombosis may be negligible, if there is good flow after SCB usage.

When interpreting our results, one needs to consider that we aim for vigorous lesion preparation, including cutting and/or non-compliant balloons at high pressure, in the majority of DCB cases treated at our site (16, 22, 23). The importance of such a strategy in order to achieve good long-term outcomes following DCB treatment has been shown before (5, 16, 22, 23). We also liberally use intravascular imaging, namely OCT, in DCB cases, which not only facilitates PCI device selection and DCB sizing, but also the assessment of luminal gain as well as dissections at the end of DCB treatment (16).

To what extent the crystalline sirolimus coating had an impact on our studýs outcomes, remains somewhat uncertain and it will require more studies, including randomized head-to-head comparisons with other DCBs, to answer this question. Nonetheless, the crystalline sirolimus coating showed a distinguished drug transfer rate and metabolism in the vessel wall after application in experimental studies (8). Also, the studied crystalline SCB carries a higher drug-concentration than other available SCBs (4.0 μg/mm^2^ vs. <1.5 μg/mm^2^), which could be important to achieve a lasting cytostatic effect and thus meaningful inhibition of neointima hyperplasia (5).

Yet, it is not possible to draw any firm conclusions, as to whether sirolimus should become the mainstay for DCBs (11). Of note, there is an ongoing prospective multicenter single-arm trial (SCORE trial, NCT04470934) evaluating the safety and outcomes following treatment with the novel crystalline SCB SeQuent® (12). This trial plans to enroll more than 1,000 patients and will certainly provide more important insights about the performance of this DCB (12).

The concept of stent- or implant-free PCI represents an appealing and modern therapeutic option for a large portion of CAD patients undergoing PCI. Sirolimus, as a cytostatic limus-drug, may not only have greater therapeutic window, but it may provide several other advantageous effects on the vessels in comparison to the cytotoxic drug paclitaxel (7, 11, 12). In fact, paclitaxel has been shown to lead to cell necrosis, which can consequently leave the vessel wall permanently injured (24). With regards to the DES data, sirolimus and its derivates have a more pronounced anti-restenotic effect, including anti-inflammatory properties, which could become important on the long run (7, 11, 12).

Our study has several limitations. First, this is an observational single-center study, which may limit its generalizability. Also, we lack a comparator cohort. For those reasons, our data should be considered as hypothesis generating only. Second, our studýs sample size is relatively small, which limits statistical power. Also, we lost 7 (6%) patients during follow-up and follow-up rate was not possible for all patients at 1 year. This could have influenced the MACE rate. Third, follow-up assessment was only possible by phone calls in some cases enrolled in our study. However, we are well aware that routine clinical and moreover imaging follow-up would have provided more detailed insights about the performance of this crystalline SCB. Fourth, >80% of all individuals enrolled in this study were males. Thus, it remains somewhat uncertain, if our also translate to females managed with this SCB. Finally, it would be important to have extended follow-up data (beyond 2 years) to make conclusions about the long-term safety and outcomes of CAD treatment with this crystalline SCB.

## Conclusion

Our data suggests promising outcomes following treatment using this novel SCB in all-comers. Our data showed a high rate of procedural success (e.g., no acute vessel closure), and a low rate of MACE at 1 year follow-up (<5%). Nonetheless, these results require further confirmation with dedicated randomized trials.

## Data Availability

The raw data supporting the conclusions of this article will be made available by the authors, without undue reservation.
